# Cluster headache in Greece: an observational clinical and demographic study of 302 patients

**DOI:** 10.1186/s10194-016-0683-0

**Published:** 2016-09-26

**Authors:** Michail Vikelis, Alan M. Rapoport

**Affiliations:** 1 Headache Clinic, Mediterraneo Hospital, Glyfada, Greece; 2 Glyfada Headache Clinic, 8 Lazaraki Str, Glyfada, 16675 Greece; 3 The David Geffen School of Medicine at UCLA, Los Angeles, California USA

**Keywords:** Cluster headache, Episodic cluster headache, Chronic cluster headache, Cluster headache phenotype, Cluster headache demographics, Error, Delay, Misdiagnosis, Mismanagement, Mistreatment

## Abstract

**Background:**

Cluster headache (CH) is considered the most excruciating primary headache syndrome; although much less prevalent than migraine, it is not rare as it affects more than 1/1000 people. While its clinical presentation is considered stereotypic, atypical features are often encountered. Internationally, cluster headache is often misdiagnosed, undertreated and mistreated.

**Methods:**

We prospectively studied 302 CH patients, all examined by the same headache specialist. The aim of our study was to describe the demographic and clinical characteristics of CH patients in Greece and draw attention to under-management, under-treatment and mis-treatment often encountered in clinical practice; our purpose is to improve recognition and successful treatment of cluster patients by Greek neurologists and other physicians.

**Results:**

In the present cohort, clinical characteristics of CH are similar to those described in other populations. Beyond the standard clinical characteristics, features like side shifts (12.6 %), location of maximal pain intensity outside the first trigeminal branch division (10.2 %), lack of autonomic features (7 %), presence of associated features of migraine and aggravation by physical activity (10 %) were encountered. Four out of five patients had consulted a physician prior to diagnosis. The median number of physicians seen prior to diagnosis was 3 and the median time to diagnosis was 5 years, though it improved for patients with recent onset. Chronic cluster headache, side shifts, pain location in the face or the back of the head and aggravation by physical activity were found, among others, to be statistically significantly related to delayed diagnosis or more physicians seen prior to diagnosis. Even properly diagnosed patients were often undertreated or mistreated.

**Conclusions:**

Cluster headache, in a large cohort of Greek patients, has the same phenotypic characteristics as described internationally. Uncommon clinical features do exist and physicians should be aware of those, since they may eventuate in diagnostic problems. Most CH patients in Greece remain misdiagnosed or undiagnosed for rather lengthy periods of time, but time to diagnosis has improved recently. Even after diagnosis, treatment received was suboptimal.

## Background

Cluster headache (CH), the most common trigeminal autonomic cephalalgia, is considered to be the most excruciating form of primary headache, causing profound ictal disability to sufferers [1, 2]. Although cluster headache was described long ago by a variety of names, systematic study of its clinical characteristics and significant advances in understanding its pathophysiology, began taking place only in the last 40–50 years, pioneered by the work of Dr. Lee Kudrow [3, 4]. He studied oxygen inhalation therapy as a way to abort attacks; the importance of light and flying through time zones as triggers; and predicted that the hypothalamus was a critically important area of the brain involved in its pathophysiology [3, 4]. From a clinical, pathogenic and therapeutic point of view, CH is distinct from other primary head syndromes such as migraine [1–6]. Cluster headache attacks are stereotypical and the usual clinical characteristics include side-locked pain episodes that are accompanied by ipsilateral cranial autonomic symptoms, most often lacrimation, conjunctival injection and rhinorrhea. The attacks are relatively short-lasting, usually 45–90 min and may differ in duration, frequency, intensity of pain and presence of autonomic symptoms [1–7]. Cluster headache is well described and characterized within the International Classification of Headache Disorders and may be diagnosed as episodic or chronic, the latter one characterized by CH attacks for more than a year with no remission or with remission for less than a month [8]. The disorder is more prevalent in men, although the male to female varies across studies [9].

Although often described by the word “rare” [10], CH is not actually rare, as its prevalence in population-based samples is estimated to be about 1/1000 [9], which is comparable to that of multiple sclerosis [11]. Still, worldwide, CH is considered to be largely underdiagnosed and undertreated, as a significant percentage of CH patients are not treated according to existing guidelines or evidence-based treatment recommendations [12, 13].

Characteristics of CH patients in various countries [7, 14–17] and diagnostic and therapeutic trends and issues [7, 18–21] have been described in a number of publications, but so far no epidemiologic data have been published from Greece. We prospectively studied a large number of CH patients visiting two specialized headache clinics, located in Glyfada, Greece, and examined by the same headache specialist. The aim of our study was to describe the demographic and clinical characteristics of CH patients in Greece; in so doing, we hoped to draw attention to under-management and under-treatment often encountered in clinical practice, with the purpose of improving recognition and successful treatment by Greek neurologists and other physicians.

## Methods

This study was a prospective clinical study, conducted in accordance with the principles of the Helsinki Declaration. Written informed consent was obtained from every patient. The study was approved by the first author’s Institutional Review Board (Mediterraneo Hospital, protocol no. 1640). Data on consecutive CH patients (*n* = 302) were prospectively recorded in Glyfada Headache Clinic and in Mediterraneo Hospital Headache Clinic from February 2007 until June 2015. Patients came from all geographical regions of Greece, mainly through self-referral (84.7 %), although we did get referrals from other physicians, including neurologists. All patients were examined by the same headache specialist (MV). A detailed history was obtained through a semi-structured questionnaire, a complete neurological examination was performed, further examinations assessed or requested when needed and patients were finally assigned a diagnosis according to the International Headache Society (IHS) diagnostic criteria. After the publication of the last version of the International Classification of Headache Disorders (ICHD-III beta) [8], all previous cases were revisited and the IHS-III beta criteria were used for reconfirming diagnosis. All data were inserted in a database, double-checking for typing errors. Only data from patients diagnosed with cluster headache according to the IHS-III beta criteria (digit codes 3.1.1 and 3.1.2) were entered into the database and further analyzed. X2 test of independence or Fisher’s exact test was used to evaluate the association between two qualitative variables. Kruskal-Wallis or Mann-Whitney test was used to evaluate the association between groups and the continuous characteristics of the sample (eg. age, etc.). Nominal variables are presented in absolute and relative (%) frequencies, while continuous variables are presented by median and range or mean and standard deviation. The 5 % significance level (alpha = 0.05) was considered as statistically significant. All statistical analyses were performed with version 20 of the SPSS package (SPSS Inc, Chicago, Il, USA).

## Results

### Demographics and CH types

Our cohort is comprised of 302 Greek patients with cluster headache. The mean duration of follow up was 28.4 months. As expected, CH was more common in men than in women; 237 of our patients (78.5 %) were men while 65 (21.5 %) were women, resulting in a male-to-female ratio of 3.6:1. Most patients were diagnosed with episodic cluster headache (ECH) (234/302, 77.5 %). Chronic cluster headache (CCH) was diagnosed in 68/302 (22.5 %). Women accounted for 21.4 % in the ECH group and 22.1 % in the CCH group, resulting to a similar male-to-female ratio in both groups. The male to female ratio was found to decrease with the decade of onset, from 6:1 for onset before 1989, to 4.3:1 for onset between 1990 and 1999, 4.2:1 for onset from 2000 to 2009 and 2.3/1 for onset since 2010, though this change did not reach statistical significance (*p* = 0.139) (Table 1).

**Table 1:** Male to female ratio by decade of onset

Decade of onset	Males (*N* = 237)	Females (*N* = 65)	Male to female ratio
<1989	26	4	6
1990–1999	56	13	4.3
2000–2009	100	24	4.2
2010-present	55	24	2.3

### CH family history and social habits

A positive family history of CH was reported by 17.5 % of patients (16.7 % in ECH and 20.6 % in CCH) and in most cases involved a parent with similar symptoms (usually undiagnosed, but with typical witnessed clinical description). Smoking was quite common, with the majority of our patients reporting being current (61.3 %) or past smokers (12.9 %). Current alcohol consumption was reported by 58.3 % of the patients, while 16.9 % of them were former alcohol users. Alcohol was reported as a trigger for CH attacks by 84,8 % of males and 45,3 % of females (*p* = 0.001). Use of recreational illegal substances was reported by 14.9 % as current and by 15.6 % as prior. Most of CH patients in our series worked on a regular basis (or were full-time students) (63 %), but 21 % were unemployed and 16 % worked occasionally.

### CH onset and course

The median age at onset of CH in our series was 29 years of age (mean: 32 years), with the youngest patient reporting disease onset at the age of 8 and the oldest at the age of 67 (both were ECH patients). In the ECH group, median age of onset was 29 years (mean 31.5 years), while in the CCH group, the median age of onset was 30 years (mean 33.2 years). The youngest and oldest age at onset was 8 and 68 years for men and 12 and 67 years for women, respectively. In CCH the youngest reported age of onset was 13 years and the oldest was 66 years of age. In ECH, the vast majority started as episodic, but 8 patients out of 234 (3.3 %) evolved from an initially chronic form. In CCH, 50 out of 68 patients (73.5 %) patients experienced CCH since onset, while in 18 of them (26.5 %) CH evolved from an initially episodic form.

### Clinical features

#### Pain location

In our cohort, pain was reported to localize in various areas, including the orbital, supraorbital and temporal area, but also the occiput, the upper and lower teeth, the jaw, the nose, the cheek, the neck, the ear, the shoulder and the vertex (Table 2). Still, the orbital, supraorbital and temporal areas were most commonly involved (in 91.7, 56.6, and 75.5 % of patients, respectively) with 48 % of our patients reporting pain in all three of these areas. It is worth mentioning that 5 patients were excluded from this analysis as they reported no pain in any of these three areas and as such they did not fulfill criterion B of the International Headache Society [8]. They were otherwise typical CH patients. The majority of our patients reported maximal pain intensity in the orbital, supraorbital and temporal area (68.5, 7.3 and 13.6 %, respectively), as typically described. It should be noticed however that additional areas, rather atypical for maximal pain intensity, have been occasionally reported, such as the occiput (4 % of the group), the vertex (1 % of the group), the upper teeth (2 % of the group) and the cheek (2.6 % of the group), while 2 patients had maximal pain intensity in the neck and one in the jaw. No significant differences in the areas involved were noticed between men and women, except for the temporal area, which was more frequently involved in men (*P* = 0.048). Finally, the neck, shoulder and vertex as pain localizations were significantly more frequently reported in the CCH group (*p* = 0.02, 0.001 and 0.01, respectively) as compared to the ECH group (Table 2).

**Table 2:** Site of pain (%, *N* = 302, unless otherwise indicated)

	Total	ECH	CCH	Men	Women
Orbital (%)	91.7	92.3	89.7	93.2	86.2
Supraorbital (%)	56.6	56.4	57.4	56.1	58.5
Temporal (%)	75.5	75.2	76.5	78.1*	66.2*
Occiput (%)	45.0	45.7	42.6	47.3	36.9
Upper teeth (%)	26.2	24.4	32.4	26.2	26.2
Lower teeth (%)	12.3	11.5	14.7	12.2	12.3
Jaw (%)	18.5	17.9	20.6	20.7	10.8
Cheek (%)	24.2	21.8	32.4	26.6	15.4
Nose (%) (*N* = 299)	10.0	9.5	11.8	10.6	7.8
Ear (%) (*N* = 301)	10.0	9.9	10.3	9.3	12.5
Neck (%)	15.9	13.2*	25.0*	16	15.4
Shoulder (%) (*N* = 300)	6.7	3.9*	16.2*	7.2	4.7
Vertex (%) (*N* = 293)	24.7	21.5*	36.9*	23.9	28.6

*Indicates statistically significant difference between two groups (see text for exact *p* value)

#### Side of pain, laterality, side shifts

While the majority of our patients experienced strictly unilateral pain (87.4 % of total, right sided 47.0 % and left-sided 40.4 %) without a side shift, 12.6 % reported at least one type of a side shift, most commonly a side shift between bouts (9.0 %), followed by side shift within bouts (3.0 %) and side shift within attacks (1.7 %). In the subgroup of episodic cluster headache, 0.9 % had experienced a side shift within an attack, 2.1 % had experienced a side shift within a bout and 10.4 % had experienced a shift between bouts (shift to the other side at the beginning of a new bout). In the CCH group, side shift within attacks was reported by 4.4 % of patients, while side shift within bouts was reported by 5.9 % and side shift between bouts by 4.4 % of patients (when periods of remission were reported in the history of a CCH patient, Table 3). One single patient that evolved from episodic to chronic cluster headache later in the course of his disease developed a side shift between attacks and subsequently a side shift within attacks. During the within-attack side shifts, the pain was reported to be simultaneously present in both the right and left orbital and supraorbital area, at high intensity, accompanied by bilateral autonomic features, a characteristic that has been previously reported in other CCH patients [17]. Side shift within attacks and within bouts was more frequently reported in the CCH than in the ECH group while any type of side shift was more common in men that in women, although the differences identified did not reach statistical significance (Table 3).

**Table 3:** Side of pain, laterality, side shifts (%, *N* = 302, unless otherwise indicated)

	Total	ECH	CCH	Men	Women
Strictly unilateral (%)	87.4	86.3	91.1	86.4	90.8
Strictly left (%)	40.4	39.7	42.6	40.9	38.5
Strictly right (%)	47.0	46.6	48.5	45.6	52.3
Side shift between bouts (%) (*N* = 299)	9.0	10.4	4.4	9.8	6.2
Side shift within bouts (%) (*N* = 301)	3.0	2.1	5.9	3.4	1.5
Side shift within attacks (%)	1.7	0.9	4.4	2.1	0.0

### Autonomic and associated features, character of pain

The most commonly reported autonomic feature was lacrimation, followed by nasal congestion/rhinorrhea and conjunctival injection (detailed percentages in Table 4). It should be noted that 21 patients (7 % of total) in our cohort had none of the autonomic features listed in the criterion C1 of the ICHD-III beta [8]. Absence of autonomic features was more prevalent in women that in men (4.6 % of males vs. 15.4 % of females had no autonomic features, *p* = 0.003). Patients with no autonomic features tended to have disease onset at an older age than those with at least one autonomic feature (median age of disease onset 43 vs. 29 years, but this difference did not reach statistical significance; *p* = 0.064). In between sex comparison, lacrimation and conjunctival injection were less frequently reported in women than in men (*p* = 0.031 and *p* = 0.026, respectively), while no differences were noticed between ECH and CCH. It is also worth mentioning that autonomic features added in criterion C of ICHD-III beta for CH, namely ipsilateral sensation of fullness in the ear and ipsilateral forehead/facial flushing, were rather commonly reported in our group, by 28.1 and 27.1 % of patients, respectively (data available only for a third of total group) (Table 4). Despite that, there was not a single case of a patient without presence of any of the autonomic features included in the ICHD-II (conjunctival injection and/or lacrimation, nasal congestion and/or rhinorrhea, eyelid oedema, forehead and facial sweating) in whom forehead and facial flushing or sensation of fullness in the ear was reported. Associated features considered typical for migraine but often disregarded in CH were also quite common (Table 5). Among them, photophobia was the most prevalent (61.6 %) in our cohort and, when present, in most patients it was reported to be lateralized to the suffering side. Phonophobia, nausea and vomiting were also reported (Table 5). In addition, yawning and belching just preceding or during the final phase of an attack were reported by approximately one out of five patients. Another CH characteristic that is included in the IHS classification criteria, a sense of restlessness or agitation during attacks, was reported by 83.4 % of our patients and was less common in women than in men (75 % vs. 85.7 %, *p* = 0.042). However, It should be noted that 10.0 % of our group reported pain aggravation by physical activity and the majority of them preferred to stand still or lay down during attacks. This represents a quite atypical feature, compared to classical cluster headache descriptions. The character of pain, as expected, was described as lancinating or stabbing in the vast majority of patients, followed by throbbing or pressing pain (Table 5). Patients with CCH had a lower percentage of stabbing/lancinating pain (82.4 %) in comparison to those with ECH (90.6 %) and a higher percentage of throbbing pain (10.3 % vs. 6.4 %) and any other kind of pain (mainly burning pain) (7.4 % vs. 1.3 %) (*p* = 0.019 for these comparisons).

**Table 4:** Autonomic features (%, *N* = 302, unless otherwise indicated)

	Total	ECH	CCH	Men	Women
Lacrimation (%)	82.8	83.3	80.9	85.2*	73.8*
Conjunctival injection (%)	66.9	64.5	75.0	70.0*	55.4*
Nasal congestion and/or rhinorrhea (%)	77.1	78.1	73.5	78.8	70.8
Eyelid oedema (%)	35.4	35.5	35.3	36.7	30.8
Forehead and facial sweating (%)	19.2	20.5	14.7	198	16.9
Miosis and/or ptosis (%)	34.6	34.8	33.8	36.4	27.7
None of the above (%)	7.0	6.4	8.8	4.6*	15.4*
Forehead and facial flushing (%) (*N* = 133)	27.1	26.9	27.6	26.9	27.6
Sensation of fullness in the ear (%) (*N* = 128)	28.1	28.3	27.6	27.3	31.0

*Indicates statistically significant difference between two groups (see text for exact *p* value)

**Table 5:** Associated features and character of pain (%, *N* = 302, unless otherwise indicated)

	Total	ECH	CCH	Men	Women
Photophobia (%)	61.6	60.3	66.2	61.6	61.5
Phonophobia (%)	36.8	34.6	44.1	38.0	32.3
Nausea (%)	22.8	22.2	25.0	23.6	20.0
Vomiting (%) (*N* = 301)	7.0	6.9	7.4	7.6	5.7
Belching (%) (*N* = 300)	21.0	21.0	20.9	22.0	17.2
Yawning (%)	19.5	18.4	23.5	21.1	13.8
Sense of restlessness or agitation (%) (*N* = 301)	83.4	82.8	85.3	85.7	75.0
Pain aggravation by physical activity (*N* = 301)	10.0	9.9	10.3	8.5	15.4
Stabbing/lancinating pain (%)	88.7	90.6*	82.4*	89.9	84.6
Throbbing pain (%)	7.3	6.4*	10.3*	7.6	6.2
Pressing pain (%)	1.3	1.7	0.0	1,3	1.5
Other type of pain (%)	2.6	1.3*	7.4*	1.3	7.7
Attacks triggered by alcohol (*n* = 301)	76.4	74.7	82.4	84.8*	45.3*

*Indicates statistically significant difference between two groups (see text for exact *p* value)

#### Attack duration and frequency, bout duration, periodicity

Some characteristics of CH, including attack duration and frequency, may vary within the same patient during a cluster period. Shorter or less frequent attacks may be encountered at the beginning or ending of a bout and longer and more frequent attacks at the peak. in order to better describe the clinical features, we recorded not only the usual or typical duration or frequency of attacks but also the usual minimum and maximum values for those. The median usual duration of attacks was 60 min for both ECH and CCH patients, while the median usual minimum attack duration was 40 min (45 for ECH and 30 for CCH, respectively, *p* = 0.009), and the median usual maximum attack duration was 90 min (for both ECH and CCH). It should be noted that in some patients maximum attack duration in individual attacks exceeded 180 min and reached up to 420 min (Table 6). The median number of attacks per day was 1 (1 for ECH and 2 for CCH, *p* = 0.001), while the median usual maximum number of attacks per day was 3, 2 and 3.5 for total group, ECH and CCH, respectively (*p* = 0.001 for ECH vs. CCH). The median number of bouts per year was 1 (range: 0.1–6). The median usual duration of bouts for ECH was 4 weeks (range: 1–24 weeks). Seasonal propensity of bout onset for ECH was reported by 46.2 % patients in our cohort, while a daily propensity of attack onset (mainly nocturnal) was reported by 47 % of total cohort and was significantly more common in ECH than CCH (53.8 % vs. 23.5 %, *p* = 0.001).

**Table 6:** Attack duration and frequency

	Total group		ECH	CCH	Males	Females
	Median (range)	Mean (SD)	Median (range)	Median (range)	Median (range)	Median (range)
Usual attack duration (min)	60 (20–180)	66.7 (35.3)	60 (20–180)	60 (20–180)	60 (20–180)	60 (25–180)
Usual minimum attack duration (min)	40 (5–180)	44.3 (30.5)	45* (10–180)	30* (5–90)	40 (5–180)	30 (10–180)
Usual maximum attack duration (min)	90 (30–420)	109.1 (67)	90 (30–420)	90 (30–300)	90 (30–420)	90 (30–300)
Usual attack frequency/24 h	1 (1–6)	0.9 (0.6)	1* (0.5–6)	2* (0.3–8)	1 (0.3–8)	1 (0.5–6)
Usual maximum attack frequency/24 h	3 (0.5–10)	2.9 (1.7)	2* (1–10)	3,5* (0.5–9)	3 (1–9)	3 (0.5–10)
Usual bout duration (weeks)	6 (1–52)	16.1 (19)	4 (1–24)	-	7 (1–52)	6 (1–52)
Usual minimum bout duration (weeks)	4 (0.5–52)	12.8 (17)	4 (0.5–24)	-	4 (1–52)	4 (1–52)
Usual maximum bout duration (weeks)	8 (0–52)	16.6 (17.7)	8 (0–50)	-	8 (1–52)	8 (1–52)

*Indicates statistically significant difference between two groups (see text for exact *p* value)

### Diagnostic and therapeutic issues

In the majority of our patients (175/302) a diagnosis of CH had not been previously made and was established during consultation at our Center for the first time. Among the 127 previously diagnosed patients, most of them were diagnosed by a neurologist (70.2 %), followed by a primary care physician, neurosurgeon and ENT specialist, (5.6, 4.8, 3.2 % respectively). Overall, 87.4 % of patients were diagnosed by a neurologist, including patients diagnosed at our clinic. Interestingly, 39 cases (12.9 % of total), reported a self-diagnosis, primarily through information found on the internet, with subsequent medical confirmation. In the majority of these cases, one or more erroneous diagnoses had been made by physicians previously. Hence, self-diagnosis was the second more common way of diagnosis, a fact indicating the importance of medical information provided to public. The median time from disease onset to diagnosis in our cohort was 5 years (range 0–45, mean 7.2 years). Overall, time to diagnosis significantly improved with decade of onset, for the current decade being just one year (median), compared to 5 years for the 2000s, 12 years for the 1990s and 20 years for onset before 1990 (Table 7). This also applied to all subgroups (males, females, ECH and CCH) (*p* = 0.001 for all comparisons between subsequent decades, applies also to all subgroups).

**Table 7:** Years from disease onset to diagnosis, correlated to decade of onset

	Tota Median (range)	ECH Median (range)	CCH Median (range)	Men Median (range)	Women Median (range)
Decade of onset of CH					
< 1989	20 (0–45)	18 (0–45)	30 (20–30)	18 (0–41)	23 (20–45)
1990–1999	12 (2–21)	11 (2–21)	13 (2–16)	12 (3–21)	12 (2–16)
2000–2009	5 (0–14)	5 (0–14)	5 (0–12)	5 (0–12)	3 (0–14)
2010-present	1 (0–7)	1 (0–7)	1 (0–6)	0 (0–6)	3 (0–7)

*p* = 0.001 for all comparisons between subsequent decades, applies also to comparisons between subgroups

In 245/302 patients (81.1 % of total group), medical consultation was sought prior to the diagnosis of CH. In more detail, before being diagnosed, 63.5 % of the total cohort had consulted a primary care physician (general practitioner or internist, in Greece), 35.5 % an ENT specialist, 31.4 % an ophthalmologist, 25.9 % a dentist, 41.3 % a neurologist and 8.5 % a neurosurgeon, without having received a diagnosis of CH. Half the patients in our cohort (51 %) had been diagnosed as migraineurs prior to CH diagnosis, 42.3 % as suffering from trigeminal neuralgia, 11 % as suffering from ophthalmic disease, 25.1 % received an ENT disease diagnosis, 15.1 % a dental problem/temporomandibular joint disorder diagnosis and 12 % a cervical spine disorder diagnosis (Table 8). The median number of physicians seen prior to diagnosis was 3 (range 0–15, mean 3.5) and significantly improved with decade of onset, from a median of 7 doctors seen prior to diagnosis for onset before 1989 to a median of 5, 3 and 1 for onset between 1990–1999, 2000–2009 and after 2009, respectively (*p* = 0.001 for all comparisons). Patients with CCH had seen a larger number of physicians prior to diagnosis than ECH sufferers (median 4 vs. 2, *p* = 0.0001), while number or physicians prior to diagnosis did not differ between males and females (median 3, in both cases).

**Table 8:** Diagnoses made and physicians seen prior to CH diagnosis

A. Diagnoses made prior to CH diagnosis (%)		B. Physicians seen prior CH to diagnosis (%)	
Migraine	51.0	Primary care physician	63.5
Trigeminal neuralgia	42.3	Dentist	25.9
Ophthalmic disease	11.0	ENT specialist	35.5
Dental or jaw disease	15.1	Ophthalmologist	31.4
ENT disease	25.1	Neurologist	41.3
Cervical spine disease	12.0	Neurosurgeon	8.5
		Other	22.6

Although time to diagnosis and number of physicians seen prior to diagnosis improved over time, we further investigated whether certain disease characteristics that were encountered less often or features that are considered atypical resulted in diagnostic delays. Factors identified as significantly correlated with greater number of years lapsed to diagnosis included earlier decade of onset, presence of side shift between bouts, pain location in the jaw, cheek, lower teeth or ear area, presence of photophobia, forehead and facial sweating, pain aggravation by physical activity and absence of typical cluster headache autonomic features (exact *p* values presented at Table 9). In addition, factors associated with a greater number of physicians prior to diagnosis included presence of CCH, earlier decade of onset, pain location in upper teeth, cheek, lower teeth, neck, nose, ear, shoulder or vertex, presence of eyelid oedema, miosis/ptosis and aggravation by physical activity (exact *p* values presented at Table 10). Furthermore, increased usual and maximum duration of bouts and increased usual and maximum attack frequency were also correlated with seeing a larger number of physicians prior to diagnosis, though these correlations were weak (Table 10).

**Table 9:** Factors correlated with more years to diagnosis

	Years to diagnosis	
	Median (Range)	*p*-value
Decade of onset		0.001
< 2000	13 (0–45)	
2000–2009	5 (0–14)	
≥ 2010	1 (0–7)	
Side shift between bouts		0.008
No	5 (0–45)	
Yes	8 (0–26)	
Jaw location of pain		0.002
No	5 (0–30)	
Yes	7 (0–45)	
Cheek location of pain		0.015
No	5 (0–30)	
Yes	7 (0–45)	
Lower teeth location of pain		0.015
No	5 (0–30)	
Yes	10 (0–45)	
Ear location of pain		0.041
No	5 (0–41)	
Yes	10 (0–45)	
Photophobia		0.016
No	4 (0–30)	
Yes	6 (0–45)	
Aggravation by physical activity		0.008
No	3 (0–20)	
Yes	6 (0–45)	
Forehead and facial sweating		0.018
No	5 (0–30)	
Yes	6 (0–45)	
Absence of autonomic features		0.023
No	2 (0–14)	
Yes	5 (0–45)

**Table 10:** Factors correlated with seeing increased number of physicians prior to diagnosis

	Median (Range)	*p*-value
Number of physicians		
CH subtype		0.001
ECH	2 (0–15)	
CCH	4 (0–15)	
Decade of onset		0.001
< 2000	5 (0–15)	
2000–2009	3 (0–11)	
≥ 2010	1 (0–10)	
Upper teeth location of pain		0.001
No	3 (0–15)	
Yes	4 (0–15)	
Jaw location of pain		0.001
No	3 (0–15)	
Yes	4 (0–15)	
Cheek location of pain		0.001
No	2 (0–15)	
Yes	5 (0–15)	
Lower teeth location of pain		0.002
No	3 (0–15)	
Yes	5 (0–15)	
Neck location of pain		0.001
No	3 (0–15)	
Yes	5 (0–15)	
Nose location of pain		0.007
No	3 (0–15)	
Yes	5 (0–15)	
Ear location of pain		0.001
No	3 (0–15)	
Yes	5 (1–15)	
Shoulder location of pain		0.001
No	3 (0–15)	
Yes	8 (1–11)	
Vertex location of pain		0.036
No	3 (0–15)	
Yes	4 (0–10)	
Aggravation by physical activity		0.024
No	2 (0–10)	
Yes	3 (0–15)	
Eyelid oedema		0.024
2 (0–10)	3 (0–15)	
2 (0–10)	4 (0–15)	
Miosis and/or ptosis		0.001
2 (0–10)	2 (0–15)	
2 (0–10)	4 (0–15)	
Spearmans’ rho		
Usual maximum duration of bout	0.208	0.001
Usual duration of bout	0.224	0.001
Usual attack frequency	0.220	0.001
Usual maximum attack frequency	0.310	0.001

Cluster headache patients can be treated with evidence-based or guideline-recommended treatments [10, 13, 22–26], but mistreatment is often reported [19–21] and treatments that are proven as not effective, as in the case of indomethacin [27] or single high dose steroids [28], or have unconfirmed efficacy, as in the case of octreotide [29] are internationally often used. Use of inappropriate treatments or procedures or lack of recommendation of appropriate treatment was often encountered in our cohort, not only in undiagnosed patients but, importantly, also in patients previously diagnosed. Among the total group, 188 patients (62.7 %) had received pharmaceutical treatment of any type prior to CH diagnosis and 42 patients (14.0 %) had undergone unnecessary procedures, mainly by dentists (10.2 %) and ENT specialists (9.9 %), most commonly tooth extractions, fillings, sinus washout or surgery for nasal septum deviation, in all cases without success. In a single patient with severe cheek/ jaw pain, all teeth on the upper jaw ipsilateral to pain had been extracted. Among the 127 previously diagnosed patients, only a minority had been offered treatment with subcutaneous sumatriptan or high flow oxygen for acute attacks or verapamil, corticosteroids or lithium for prevention (Table 11). In addition, a substantial proportion was offered treatment with carbamazepine, flunarizine, antidepressants or alternative treatments. Use of recommended treatments, such as sc sumatriptan, O2 inhalation, corticosteroids or verapamil did not seem to be much more common even among previously diagnosed patients who had been diagnosed by a neurologist (Table 11).

**Table 11:** Treatments offered to previously diagnosed patients, (%)

	Overall % (*N* = 127)	Previously diagnosed by a neurologist % (*N* = 89)
Subcutaneous sumatriptan	2.4	3.4
O2 inhalation	13.5	13.5
Triptans orally	31.0	33.7
Corticosteroids orally	43.7	44.9
Verapamil	18.3	20.2
Lithium	3.2	2.2
Topiramate	23.8	23.6
Valproate	6.3	6.7
Carbamazepine	18.3	15.7
Flunarizine	16.7	19.1
Tricyclic antidepressants	10.3	9.0
SSRIs, SNRIs	11.1	10.1
Alternative treatments	28.2	32.2

## Discussion

Although less common than other primary headaches, cluster headache is not rare per se [9, 12, 13]. Still, worldwide it is reported to remain largely under-recognized and sub-optimally or poorly treated both in primary care and by specialists, despite the fact that its severity would warrant prompt diagnosis and treatment [7, 12, 18–21, 30]. Therefore, it is critical that all physicians be familiar with diagnosing and managing cluster headache. In order to improve recognition and successful treatment of CH by Greek neurologists and general physicians, we undertook the task of describing the demographic, clinical characteristics, diagnostic and treatment paths of a large cohort of CH patients, seen over a lengthy period of time by the same headache specialist.

Overall, our data are in line with previously published data from different countries [5, 7, 14–17, 31]. As expected, the typical presentation of cluster headache in Greece is indisputably stereotyped and distinct from other primary headache syndromes. Still, our data show that CH is not a male disorder, as the female to male ratio for patients with disease onset during this decade has fallen to 2.3:1 from 6:1 twenty years ago, a finding that has been noticed long ago [32]. Clinical presentation of the disease was largely similar in both genders.

Features often considered uncommon or atypical were encountered in a significant percentage of patients in our cohort. Those included side shifts, maximal pain intensity outside the first division of the trigeminal nerve, lack of all autonomic features or lack of a sense of restlessness or agitation, as other studies have also shown [5, 7, 16, 33]. Some of the atypical features, including side shifts, pain located in jaw, cheek, teeth, shoulder, vertex or neck area, were found in our study to be coexistent with, and maybe the cause of, delayed diagnosis and/or a larger number of physicians being consulted prior to diagnosis. In addition, migraine associated symptoms and autonomic features were often encountered, with photophobia being the most common, but not the only one reported in our series. This is in line with previously published data [7, 34, 35]. In our cohort, among migraine associated symptoms, aggravation by physical activity, reported by 10 % of our patients, was related both to delayed diagnosis and a greater number of physicians being consulted prior to diagnosis. It is worth mentioning that even though the autonomic features added in criterion C of the revised criteria of the International Headache Society (ICHD-III beta) for CH, (−namely ipsilateral sensation of fullness in the ear and ipsilateral forehead/facial flushing) were rather commonly reported in our group, there was not a single case of a patient without the presence of any of the autonomic features included in ICHD-II (conjunctival injection and/or lacrimation, nasal congestion and/or rhinorrhea, eyelid oedema, forehead and facial sweating) in whom forehead and facial flushing or sensation of fullness in the ear was reported. This supports the opinion that adding forehead and facial flushing and sensation of fullness in the ear in ICHD-III beta provides no added diagnostic value [36].

Concerning diagnostic delays, our data are in line with other published studies, which have already shown that CH patients are often misdiagnosed and mistreated [7, 19, 20, 30, 37]. In our cohort, eight out of ten CH patients have consulted at least one physician without been diagnosed and more than 40 % have consulted a neurologist. Furthermore, even in diagnosed patients, and despite the existence of evidence-based treatment options [10, 13, 22–26, 37, 38], use of guidelines or recommended treatments is quite low. These facts clearly point out the need for Continuous Medical Education in the field of cluster headache. Still, our study demonstrates that time to diagnosis is improving in Greece, something that might have to do with increasing access to specialized neurologic care and headache specialists. The role of medical information found mainly on the internet is increasingly important, as a significant percentage of patients in our group, despite having seen physicians, were actually self-diagnosed and self-referred to our center. In addition, despite these improvements, the typical sufferer still waits five years for a correct diagnosis and sees three physicians prior to it, having received one or more incorrect diagnoses, inappropriate treatments or having undergone unnecessary invasive procedures, as has also been described in other series [19–21].

This study does not come without limitations. The main one is the design of the study and the setting. It could be argued that a population-based study, resulting in a random sample of CH patients would be more appropriate and that CH patients who reach a specialist may represent an enriched population who would have atypical features or be more difficult to treat correctly. We believe our data do not point towards a highly enriched population. Patients visited us from all geographical regions of Greece, most patients came through self-referral (84.7 %), something quite common in Greece. Additionally, the majority of our cohort (57.9 %) were undiagnosed prior to visiting our clinic. Furthermore, extrapolating from the international CH prevalence data [9] to the Greek population (10.816.286 according to the last census) [39], an estimation of around 13.400 CH patients are expected to live in Greece. In this case, our sample would represent a little more that 2 % of the total CH patients in Greece.

## Conclusions

Cluster headache, studied in a large cohort of Greek patients, has the well-documented general characteristics described in all Western populations. Despite the concept of a stereotyped clinical picture, atypical features are quite often present and physicians should be aware of those, as they usually may result in diagnostic problems. Most patients remain undiagnosed for a lengthy period of time; even when diagnosed, the treatment they receive remains far from optimal. On the other hand, our data show that time to diagnosis is improving, reaching a median period of one year for patients with recent onset. Educational activities for neurologists and other medical specialties that treat headache patients are critically important and should result in better recognition and treatment of cluster headache patients.

## Abbreviations

CCH: Chronic cluster headache
CH: Cluster headache
ECH: Episodic cluster headache
